# Perceptions of psychiatric-trained nurses on integrating mental health into primary health care in Africa

**DOI:** 10.4102/hsag.v30i0.2878

**Published:** 2025-02-28

**Authors:** Busisiwe M. Febana, Mutshidzi Mulondo

**Affiliations:** 1 Department of Public Health, Faculty of Health Sciences, University of Free State, Bloemfontein, South Africa

**Keywords:** mental health services, integration, primary health care, nurses’, perceptions, healthcare services

## Abstract

**Background:**

This review mapped the perceptions of nurses on the integration of mental health services into primary health care (PHC) in low- and middle-income countries (LMICs).

**Aim:**

The study focused on perceptions related to mental health service integration to determine barriers and facilitators from the perspective of nurses.

**Method:**

A scoping review was conducted across relevant databases and search engines, resulting in the identification of 120 studies. Four studies met the inclusion criteria for synthesis.

**Results:**

The findings indicated that nurses perceived a significant lack of resources and training in mental health care. Barriers such as stigma and workload were prevalent barriers to integration. However, facilitators included support from leadership and multidisciplinary collaboration. Successful integration led to improved patient access to mental health services and reduced stigma.

**Conclusion:**

The integration of mental health services into PHC is a complex process influenced by multiple factors. Nurses play a critical role in this integration, and their insights are essential for developing effective strategies for integration. For proper integration of mental health services, it is recommended that resources and training for nurses to integrate mental health services into PHC in LMICs be increased. Addressing barriers like stigma and heavy workloads, along with support from leadership and multidisciplinary collaboration, is essential for improving patient outcomes.

**Contribution:**

This study contributes to the knowledge base on integrating mental health services into PHC, offering insights for policymakers and healthcare providers to enhance mental health care delivery in LMICs and similar settings.

## Introduction

Mental health services encompass a wide range of interventions aimed at promoting mental well-being, preventing mental disorders and providing care and rehabilitation for individuals experiencing mental health challenges. These services are vital for addressing the mental health burden that significantly affects individuals, families and communities. Mental health, a crucial component of overall health and well-being, is increasingly recognised for its significant impact on individuals and societal functioning. The World Health Organization (WHO) defines mental health as a state of well-being in which an individual realises their abilities, can cope with the normal stresses of life, work productively and contribute to their community (WHO 2021).

Globally, mental health services are often neglected despite their critical role in achieving overall health. High-income countries (HICs) have made strides in integrating mental health services into primary health care (PHC), with examples including community-based mental health programmes and telehealth initiatives in countries like Canada and the United Kingdom (Wainberg et al. 2017). However, low- and middle-income countries (LMICs), including many in Africa, face significant challenges, such as inadequate funding, limited trained personnel and persistent stigma (Rameez & Nasir 2023). For instance, in South Asia, cultural stigma and a lack of trained healthcare workers significantly hinder access to mental health services, while in Latin America, fragmented health systems complicate integration efforts (Dotsenko & Kolomiiets 2022).

Despite its importance, mental health care remains one of the most overlooked areas within healthcare delivery systems worldwide, particularly in LMICs (Hussain et al. 2018). The integration of mental health services into PHC systems is seen as a vital step towards addressing this neglect. Offering a holistic approach to health that emphasises equity and the treatment of mental disorders alongside physical health conditions (Dotsenko & Kolomiiets 2022; Wadoo et al. 2021). However, the integration of mental health services into PHC in LMICs faces numerous barriers (Rameez & Nasir 2023). Limited resources, including a lack of trained healthcare professionals and inadequate funding, hamper the delivery of effective mental health services (Wakida et al. 2018). Additionally, there is often a lack of awareness and understanding of mental health issues among healthcare providers including doctors, nurses and community health workers, as well as among the general public, leading to stigma and discrimination (Wakida et al. 2019). Furthermore, existing healthcare systems in LMICs are primarily designed to address acute physical health conditions, with little emphasis on the chronic nature of many mental health conditions (Wakida et al. 2018). These barriers necessitate a concerted effort to develop strategies tailored to the unique contexts of LMICs, focusing on training, resource allocation and public education (McInnes et al. 2022).

Nurses play a vital role in the integration of mental health services into PHC, particularly in LMICs, where there is frequently a deficiency of trained mental health practitioners (Kigozi-Male, Heunis & Engelbrecht 2023). Nurses frequently serve as the initial point of contact for patients pursuing health care, and their function in mental health care transcends conventional physical health duties, encompassing the provision of psychological support, early identification of mental health conditions and facilitation of appropriate patient care (McInnes et al. 2022). Studies demonstrate that nurses are progressively required to attend to the physical health requirements of patients with mental health disorders, given the intrinsic link between these two health dimensions (Chee, Wynaden & Heslop 2018; Happell, Platania-Phung & Scott 2013). Cheating et al. (2018) indicate that nurses proficient in performing physical health evaluations and interventions can markedly enhance health outcomes for patients with mental illnesses. This is especially significant as numerous people with mental health disorders frequently encounter concurrent physical health concerns, which can aggravate their symptoms of mental disorders (Happell et al. 2013).

This study’s focus on the perceptions of nurses towards integrating mental health services into PHC is significant for several reasons. Nurses, as frontline healthcare providers in this district, play a critical role in the delivery of health services. Understanding their perceptions can provide valuable insights into the barriers and facilitators of mental health integration. This, in turn, can inform the development of targeted interventions that address the specific needs and barriers of LMICs. Ultimately, the study aims to contribute to the broader goal of improving access to and the quality of mental health care, not only within the district but also as a model for other similar contexts in LMICs.

This scoping review, therefore, seeks to map the existing evidence on the integration of mental health services into PHC, with a specific focus on middle-income countries. By examining the barriers, opportunities, and outcomes associated with this integration, the review aims to contribute to the ongoing efforts to enhance mental health care delivery in resource-constrained settings, ensuring that mental health is recognised as an integral part of overall health and well-being.

## Aim

The study aimed to explore the perceptions of professional nurses with psychiatric experience regarding the integration of mental health services into PHC in LMICs.

### Objectives

1. To explore the perceptions of professional nurses, particularly those with relevant training or experience in mental health, on integrating mental health services into PHC.
2. To identify the barriers to the integration of mental health services into PHC.
3. To identify the facilitators to the integration of mental health services into PHC from the perspectives of professional nurses.

## Methods

### Study design

This scoping review utilised the methodological framework proposed by Arksey and O’Malley to systematically examine the literature concerning nurses’ perceptions of integrating mental health services into PHC, particularly in LMICs. The methodology encompassed several steps: defining clear objectives for the review, identifying and searching relevant literature, screening literature for inclusion, extracting and charting data from included studies, as well as synthesising and reporting the findings. The central research question guiding this review was: *What are the perceptions of professional nurses with psychiatric experience regarding the integration of mental health services into primary health care in LMICs?*

### Literature search strategies

The literature search was conducted across EBSCOhost and Google Scholar electronic databases to encompass a wide range of academic sources. The search covered publications from 2018 to 2023, reflecting a broad period to capture the evolving understanding and implementation of mental health service integration into PHC. Keywords used in the search strategy included combinations of Boolean operators ‘OR’ and ‘AND’, focusing on terms such as ‘nurses’ perceptions OR attitudes’ AND ‘mental health integration’ AND ‘primary health care’ AND ‘OR LMICs’. This approach was designed to retrieve articles that specifically addressed the study’s focus on nurses’ perceptions regarding the integration of mental health into PHC within the specified geographic context.

### Eligibility criteria

Inclusion criteria were set to encompass all primary research articles that explored nurses’ perceptions of mental health integration into PHC in LMICs. The review included studies published in English between 2018 and 2023. Excluded from the review were commentaries, editorials, and studies that did not directly address nurses’ perceptions of mental health service integration into PHC.

### Identification and selection of studies

Following a comprehensive literature search, the researchers systematically sifted through the retrieved publications to identify studies meeting the eligibility criteria. The process involved removing duplicates, screening titles and abstracts for relevance and then reviewing the full texts of potentially eligible studies for inclusion. The selection process was documented using a Preferred Reporting Items for Systematic Reviews and Meta-Analyses (PRISMA) flow diagram to ensure transparency and replicability.

### Data extraction from selected studies

Relevant data from the selected studies were extracted and organised into a standardised data extraction form. This form included fields for the author(s), publication year, study design, sample size, setting (geographical location and healthcare context), key findings related to nurses’ perceptions of mental health integration into PHC and any identified barriers and facilitators.

### Data collection

The data collection process for this scoping review followed a systematic approach. Literature searches were conducted across multiple electronic databases, including PubMed, Scopus and Google Scholar, to identify relevant studies published between 2018 and 2023. The search strategy included a combination of keywords such as ‘nurses’ perceptions’, ‘mental health integration’, ‘primary health care’ and ‘low- and middle-income countries’, with Boolean operators to refine results. Studies were screened for inclusion based on pre-determined eligibility criteria, which required the focus to be on nurses’ perceptions of integrating mental health services into PHC in LMICs. After duplicates were removed, the remaining studies underwent title, abstract and full-text screening, with the final inclusion documented using a PRISMA flow diagram.

### Data analysis

Data analysis involved a descriptive synthesis of the included studies, characterising the research according to its objectives, methodologies and findings. This approach facilitated the identification of common themes regarding nurses’ perceptions, barriers and facilitators. Comparative analysis was also undertaken where applicable to highlight variations in perceptions across different settings or contexts.

### Ethical considerations

Given the nature of a scoping review, which involves the synthesis of existing literature rather than the collection of primary data from participants, ethical approval was not required. However, the review adhered to ethical standards of research integrity and transparency.

## Review findings

### Description of the reviewed studies

The scoping review initially identified *120 studies* through comprehensive literature searches, with *12 studies* excluded after screening because of duplicates. After duplicate removal, *59 studies* were removed because of language and/or country inaccessibility. After further screening, *22 studies* were excluded because of study design: Commentaries and editorials were excluded.

**FIGURE 1:**
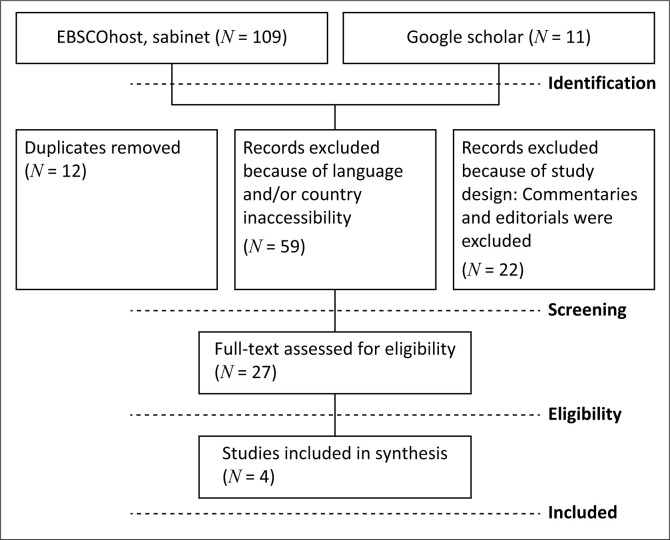
Preferred reporting items for systematic reviews and meta-analyses flow diagram.

After a full-text review, *27 studies* were assessed with *23 studies* excluded because of irrelevance or failure to meet inclusion criteria, such as not focusing on nurses’ perceptions of integrating mental health services into PHC in LMICs, leaving *4 studies* that were included in the final (see Figure 1). These studies explored nurses’ perceptions of integrating mental health services into PHC, with a focus on LMICs. These studies encompassed various research designs: one mixed-methods study, two qualitative studies and one systematic review (Ireland, Topp & Wensley 2023; Maconick et al. 2018; Mendenhall et al. 2018; Rameez & Nasir 2023). These studies focused on professional nurses, particularly those with qualifications or specialised training working in PHC (see Table 1).

**TABLE 1:** Studies utilised for low- and middle-income countries.

Authors	Publication year	Study design	Sample size	Setting	Key findings	Barriers	Facilitators	Outcomes
Ireland et al.	2023	Integrative literature review	N/A	Primary care settings, Global	Attitudinal factors play a significant role in the quality of nurse-led mental health interventions in primary care.	Nurses attitudes towards mental health interventions are influenced by stigma, lack of training and insufficient resources.	Positive workplace attitudes and organisational support improve the quality of mental health interventions.	Improvement in the quality of nurse-led mental health interventions in settings with strong support systems.
Rameez and Nasir	2023	Systematic review	N/A	Primary care settings in low- and middle-income countries (LMICs)	The study found critical gaps in mental health care in LMICs post-COVID-19, emphasizing systemic reforms to improve access and outcomes.	Inadequate infrastructure, a shortage of trained providers, high treatment costs, and persistent stigma surrounding mental health.	Enhancing provider training, increasing community awareness, leveraging digital health solutions, and strengthening policy frameworks to prioritize mental health.	The study found critical gaps in mental health care in LMICs post-COVID-19, emphasizing systemic reforms to improve access and outcomes.
Mendenhall et al.	2018	Cross-sectional study	60	Kenya (Urban and rural public and private PHC facilities)	Nurses viewed mental healthcare integration as necessary but challenging due to financial, social and system barriers.	Financial constraints, stigma surrounding mental health and limited capacity in rural areas.	Leadership support and community engagement improve perceptions of mental healthcare integration.	Integrated mental health services showed positive reception despite challenges, especially in urban settings.
Maconick et al.	2018	Quasi-experimental mixed methods study	20	South Africa (Rural primary care clinic)	In-service training improved nurses self-rated competence in mental health care.	Inadequate mental health knowledge and lack of formal training before in-service interventions.	Continuous in-service training and collaboration with local staff helped integrate mental health services.	Increased competence in mental healthcare referrals and overall care quality.

From the relevant studies, nurses generally recognise the importance of integrating mental health services into PHC but highlight several barriers (Liang, Mays & Hwang 2018). These included a lack of specialised training in mental health, insufficient resources and the stigma associated with mental health conditions among patients and healthcare workers (Carrara et al. 2019).

Major barriers identified across the studies included limited access to mental health training for nurses, lack of support from the broader healthcare system and cultural stigmas surrounding mental health (Stubbs 2014). Additionally, workload and the physical layout of PHC facilities sometimes hindered effective integration.

For example, Mendenhall et al. (2018) highlighted financial constraints and stigma as significant barriers in Kenya, where nurses felt they were not adequately prepared to provide mental health services in PHC settings. Similarly, Rameez and Nasir (2023) identified that in LMICs, barriers to mental health service integration were exacerbated in the post-COVID-19 (coronavirus disease 2019) era because of resource shortages, interrupted services and an increased burden on healthcare workers, including nurses. In South Africa, Maconick et al. (2018) noted that nurses faced challenges related to inadequate training and the physical layout of PHC facilities, which sometimes hindered effective mental health integration. Additionally, Ireland et al. (2023) emphasised that stigma and insufficient resources were attitudinal barriers influencing the quality of nurse-led mental health interventions globally.

Key facilitators mentioned were strong leadership within the PHC setting, ongoing training and education opportunities in mental health care for nurses and the development of clear guidelines and protocols for integrating mental health services. Maconick et al. (2018) found that continuous in-service training improved nurses’ self-rated competence in managing mental health cases, while Mendenhall et al. (2018) reported that community engagement and leadership support were critical in improving nurses’ perceptions of mental healthcare integration. Rameez and Nasir (2023) emphasised the importance of supportive health policies and leadership in empowering nurses through training and development, particularly in post-COVID-19 settings. Ireland et al. (2023) reinforced the positive impact of organisational support on the quality of mental health interventions.

## Discussion

### Nurses’ role and perspectives on mental health integration in primary health care

The integration of mental health services into PHC is increasingly recognised as crucial in the healthcare discourse, particularly within the context of LMICs. Nurses, operating on the front lines, are often the initial point of contact for individuals experiencing mental health issues, positioning them uniquely to initiate and influence the trajectory of mental health care within PHC. This review identified that nurses perceive significant gaps in mental health service provision, aligning with global findings. For example, Mendenhall et al. (2018) found that nurses in Kenya perceived barriers to integrating mental health care because of financial constraints, stigma and lack of resources, which is similar to the experiences of nurses in South Africa and other LMICs. Additionally, Rameez and Nasir (2023) reported that nurses in LMICs, particularly in the post-COVID-19 era, felt unprepared to meet the increasing mental health needs because of insufficient training, lack of specialised resources and the overwhelming burden placed on healthcare systems. These studies reflect the broader issue of inadequate mental health infrastructure in LMICs, which often leaves nurses feeling ill-equipped to address mental health needs effectively. This is also echoed by a study conducted by Noonan et al. (2017), which explored midwives’ perceptions and experiences of caring for women with perinatal mental health problems. The study found that midwives face several barriers when managing perinatal mental health issues, including inadequate training, lack of resources and stigma surrounding mental health, which often leaves them feeling ill-equipped to provide the necessary support. These findings parallel the experiences of nurses in PHC settings, who similarly report feeling underprepared to integrate mental health services because of insufficient mental health education and heavy workloads.

Further supporting these findings, a study by McInnes et al. (2022) on PHC nurses indicated that nurses feel inadequately trained to handle mental health conditions within PHC. This aligns with the global call for more comprehensive mental health training for healthcare providers. The lack of sufficient training and resources, coupled with the stigma surrounding mental health, remains a critical barrier to effective mental health integration in both LMICs and high-income settings (Wakida et al. 2018).

However, there are also studies that point to facilitators of successful mental health integration. For instance, Maconick et al. (2018) demonstrated that ongoing in-service training improved nurses’ confidence and competence in managing mental health cases in rural South Africa, highlighting the potential for improved mental health outcomes when resources and training are made available. Similarly, Gigaba et al. (2024) emphasised the role of supportive leadership and multidisciplinary collaboration in facilitating the integration of mental health services into PHC in resource-constrained settings. These facilitators can help mitigate some of the barriers identified in this review.

### Barriers to effective integration

Stigmatisation within healthcare and the broader community was highlighted as a key barrier to mental health integration (Carrara et al. 2019; Ordan et al. 2018; Stubbs 2014). This stigma not only affects patient outcomes but also influences healthcare workers’ willingness and ability to provide care (Vaishnav et al. 2023). A study in Lebanon supports this, noting that stigma can lead to a reluctance among healthcare professionals to work within mental health, fearing association with the stigma themselves (Abi Hana et al. 2022). Furthermore, resource constraints in the OR Tambo District mirror concerns raised in literature regarding LMICs, such as studies by Wainberg et al. (2017) and Wakida et al. (2018), which emphasise the disparity between the high burden of mental health disorders and the inadequate allocation of resources to address these issues. In agreement, Liang et al. (2018) found that inadequate health policy and a lack of intersectoral collaboration impede the scaling up of mental health services within PHC settings.

### Facilitators to integration

This review acknowledges certain facilitators that encourage mental health service integration into PHC, with supportive leadership and organisational commitment being prominent. For instance, Maconick et al. (2018) found that continuous in-service training and local leadership support significantly improved the competence of nurses in providing mental health care. Similarly, Abdulla et al. (2024) highlighted the importance of organisational commitment and multidisciplinary collaboration as critical drivers for successful mental health integration in South African PHC settings.

These facilitators align with findings from studies such as those that stress the importance of leadership in the successful implementation of integrated care models (Mendenhall et al. 2018; Rameez & Nasir 2023). This review suggests that community engagement initiatives are pivotal, resonating with the results from Duncan et al. (2023) study that advocates for the engagement of service users and community stakeholders as a means of improving mental health services. When these factors are present, there is an evident improvement in healthcare workers’ attitudes toward mental health care, paralleling the findings from a study, indicating that positive attitudes towards mental health are associated with better patient outcomes and satisfaction (Fortin, Bamvita & Fleury 2018; Melkam & Kassew 2023).

## Recommendations

Based on the review findings, several recommendations can be made. There is a clear need for more comprehensive training programmes in mental health care for nurses working in PHC settings (McInnes et al. 2022). These programmes should be accessible, ongoing and include practical components to address the specific barriers to mental health service integration. Policymakers and healthcare administrators should ensure that PHC facilities have the necessary resources and support to integrate mental health services effectively (Wakida et al. 2018). This includes sufficient staffing, physical resources and clear guidelines on mental health care integration (Abera et al. 2014). Efforts to reduce the stigma associated with mental health conditions should be intensified (Wakida et al. 2018). Community engagement programmes that involve nurses and other healthcare workers can help raise awareness about mental health and promote more inclusive attitudes towards individuals with mental health conditions, further working to reduce stigma.

## Conclusion

The integration of mental health services into PHC, especially within contexts like the OR Tambo District, is of paramount importance for addressing the comprehensive health needs of the community. This scoping review has highlighted the significant role nurses play in this integration, pointing out the myriad barriers they face, which include a lack of specialised training, insufficient resources and societal stigma towards mental health issues (Abera et al. 2014). Despite these barriers, the potential benefits of such integration – for both healthcare workers (HCWs) and the adult general population – cannot be overstated. For HCWs, addressing mental health needs within PHC settings can lead to better job satisfaction and decreased burnout by equipping them with the necessary tools and support to care for their patients comprehensively. For the general population, improved access to mental health services in PHC settings can lead to earlier detection of mental health issues, reduced stigma and overall better health outcomes.

The commonality between HCWs and the adult general population is the profound impact mental health has on their well-being, necessitating innovative solutions like digital mental health interventions, tele-counselling and peer support via social media platforms. These solutions offer a promising avenue for overcoming the barriers to mental health service integration into PHC and ensuring that individuals receive the holistic care they need.
